# Epigallocatechin Gallate Protects against Hypoxia-Induced Inflammation in Microglia via NF-κB Suppression and Nrf-2/HO-1 Activation

**DOI:** 10.3390/ijms23074004

**Published:** 2022-04-04

**Authors:** So-Ra Kim, Kyung-Joo Seong, Won-Jae Kim, Ji-Yeon Jung

**Affiliations:** Department of Oral Physiology, Dental Science Research Institute, School of Dentistry, Chonnam National University, Gwangju 61186, Korea; rjsh82@naver.com (S.-R.K.); kjseong0513@gmail.com (K.-J.S.); wjkim@jnu.ac.kr (W.-J.K.)

**Keywords:** epigallocatechin gallate, hypoxia, inflammation, microglia, NF-κB

## Abstract

Hypoxia-induced neuroinflammation in stroke, neonatal hypoxic encephalopathy, and other diseases subsequently contributes to neurological damage and neuronal diseases. Microglia are the primary neuroimmune cells that play a crucial role in cerebral inflammation. Epigallocatechin gallate (EGCG) has a protective antioxidant and anti-inflammatory effects against neuroinflammation. However, the effects of EGCG on hypoxia-induced inflammation in microglia and the underlying mechanism remain unclear. In this study, we investigated whether EGCG might have a protective effect against hypoxia injury in microglia by treatment with CoCl_2_ to establish a hypoxic model of BV2 microglia cells following EGCG pre-treatment. An exposure of cells to CoCl_2_ caused an increase in inflammatory mediator interleukin (IL)-6, inducible nitric oxide synthase (iNOS), and cyclooxygenase (COX)-2 expression, which were significantly ameliorated by EGCG via inhibition of NF-κB pathway. In addition, EGCG attenuated the expression of hypoxia-inducible factor (HIF)-1α and the generation of ROS in hypoxic BV2 cells. Furthermore, the suppression of hypoxia-induced IL-6 production by EGCG was mediated via the inhibition of HIF-1α expression and the suppression of ROS generation in BV2 cells. Notably, EGCG increased the Nrf-2 levels and HO-1 levels in the presence of CoCl_2_. Additionally, EGCG suppressed hypoxia-induced apoptosis of BV2 microglia with cleavage of poly (ADP-ribose) polymerase (PARP) and caspase-3. In summary, EGCG protects microglia from hypoxia-induced inflammation and oxidative stress via abrogating the NF-κB pathway as well as activating the Nrf-2/HO-1 pathway.

## 1. Introduction

Hypoxia or ischemia-mediated brain injury is a major cause of neurodegenerative disorders, the incidence of which has dramatically increased in recent decades [1,2]. Prolonged exposure to hypoxia in neuronal cells induces neurotoxicity, leading to neuronal cell death. However, the underlying molecular mechanism is complicated. Hypoxic ischemic brain injury occurs due to oxidative stress and inflammatory response [3]. Microglia are the major component of the immune system in the brain. They are the resident macrophages within the central nervous system and play a crucial role in regulating the inflammatory response [4]. Stress triggers microglial activation, resulting in the release of pro-inflammatory cytokines, chemokines, and reactive oxygen species (ROS). However, the excessive activation of microglia results in increased inflammatory response, leading to neuronal dysfunction, neuronal cell death and axonal degeneration. A recent study reported that activated microglia produce inflammatory mediators such as interleukin (IL)-1 beta, tumour necrosis factor-alpha (TNF-α), and nitric oxide (NO), which are linked to the pathogenesis of hypoxic injury in the brain [5]. Therefore, the inhibition of the overactivation of microglia may be potential pharmacological strategy in the treatment of hypoxia-induced brain damage.

Green tea, one of the common beverages, contains several polyphenols including significant flavonoids known as catechin; (−)-epicatechin, (−)-epigallocatechin, (−)-epicatechin gallate, and epigallocatechin gallate (EGCG) [6]. EGCG is the main polyphenolic compound underlying the health benefits of tea consumption and has the potent antioxidant properties of two triphenolic groups in its structure [7]. In addition, in vitro studies have shown that EGCG has free radical scavenging properties [8,9]. Furthermore, the antioxidant activity of EGCG demonstrated that the active site of green tea polyphenols directly reacted with oxygen free radicals are an ortho-hydroxyl group in the B ring and galloyl moiety in the C-ring [10]. On the other hand, ROSs are main factors that cause oxidative stress, which can lead to inflammation. Many studies have increasingly shown that EGCG has anti-inflammatory and anti-apoptotic effects against oxidative stress in neurons [11,12]. It was reported that EGCG increases hippocampal neural stem cells survival, including anti-oxidant effect after LPS-induced inflammation in mice [13,14]. The neuroprotective effect of EGCG on neurodegenerative diseases is related to its potential inhibitory effect on the activation of microglial cells in the brain [13,15].

Macrophages activated by external stimuli induce the activation of nuclear factor κB (NF-κB) by phosphorylating the inhibitor-κB (IκB) and its upstream kinase inhibitor-κB kinase (IKK), activator protein (AP)-1 composed of c-Fos and c-Jun, and mitogen-activated protein kinase (MAPK) signaling cascade [16]. These activated transcription factors that trigger pro-inflammatory molecules, such as IL-1, TNF-α, and nitric oxide (NO), via the induction of inducible NO synthase (iNOS) and cyclooxygenase (COX)-2 modulate the inflammatory response [17]. Moreover, the activation of anti-oxidant genes such as hemeoxygenase (HO)-1 mediated via nuclear factor (erythroid-2) related factor (Nrf)-2 could inhibit inflammatory responses in macrophages via the suppression of NF-κB activation [17].

It is important to elucidate the precise neuro-protective mechanism of EGCG. However, the effects of EGCG on inflammatory response via the hypoxia-induced activation of microglia have yet to be reported. Cobalt chloride (CoCl_2_) is widely used to establish in vitro models of chemically induced hypoxia. In the present study, we elucidated the neuroprotective effect of EGCG against CoCl_2_-induced hypoxia inflammation and the underlying molecular mechanism in BV2 microglia cells.

## 2. Results

### 2.1. Effects of EGCG on the Cell Viability and the Protective Action of EGCG on Cytotoxicity in CoCl_2_-Treated BV2 Cells

**Figure 1:**
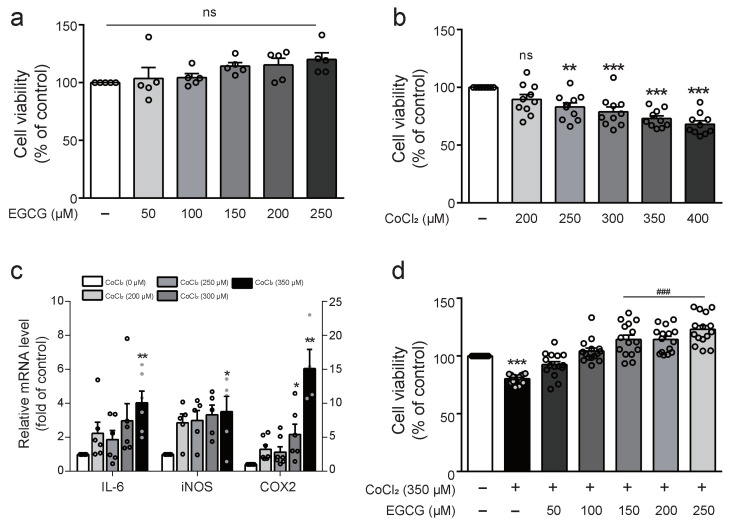
Effect of EGCG on CoCl_2_-induced cytotoxicity in BV2 cells. (**a**) Cell viability was determined by WST assay in BV2 cells in the presence of different concentrations of EGCG (50, 100, 150, 200, and 250 μM) for 8 h. (**b**) Cell viability was analysed in various concentration (200, 250, 300, and 350 μM) of CoCl_2_-treated BV2 cells by WST assay. (**c**) The mRNA expression of IL-6, iNOS, and COX-2 was analysed by real time PCR in various concentrations (200, 250, 300, and 350 μM) of CoCl_2_-treated BV2 cells. (**d**) Cells was pre-treated with or without various concentrations of EGCG (50–250 μM) for 1 h before CoCl_2_ (350 μM) exposure for 8 h and WST assay was performed. The data represent mean ± SEM based on triplicate independent experiments. ns, not significant, * *p* < 0.05, ** *p* < 0.01, *** *p* < 0.001 vs. control group; ^###^ *p* < 0.001 vs. CoCl_2_-treated group.

Cell viability was determined via a WST assay in BV2 cells cultured with various concentrations of EGCG or CoCl_2_ for 8 h. EGCG treatment resulted in no significant toxicity compared with the control, whereas CoCl_2_ significantly decreased cell viability in a dose-dependent manner (Figure 1A,B). Moreover, the expression of IL-6, iNOS, and COX-2 were upregulated significantly in cells exposed to 350 µM CoCl_2_ for 8 h (Figure 1C). Accordingly, 350 µM CoCl_2_ was treated for further experiments. The viability of cells cultured with EGCG for 1 h prior to CoCl_2_ treatment for 8 h was measured to determine the protective effect of EGCG against cytotoxicity induced by CoCl_2_ in BV2 cells. EGCG significantly restored the cell viability reduced by CoCl_2_ in BV2 cells (Figure 1D).

### 2.2. EGCG Downregulates Expression Levels of Pro-Inflammatory Cytokine and Mediators in CoCl_2_-Treated BV2 Cells

**Figure 2:**
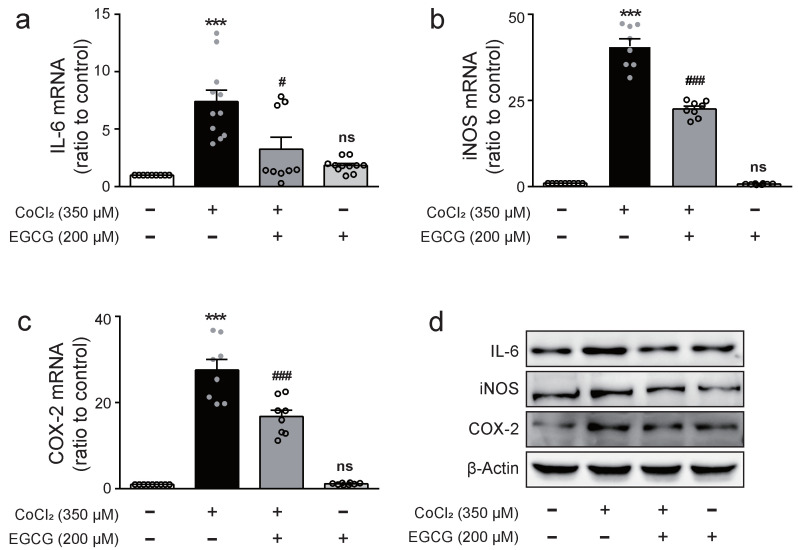
Effect of EGCG on the expression of pro-inflammatory mediators in CoCl_2_-treated BV2 cells. Cells were treated with EGCG (200 μM) for 1 h prior to CoCl_2_ (350 μM) exposure for 8 h. The mRNA expression of IL-6 (**a**), iNOS (**b**) and COX-2 (**c**) was determined by real-time PCR. (**d**) The protein expression of IL-6, iNOS, COX-2, and β-actin was detected by Western blot analysis. The data represent mean ± SEM. ns, not significant, *** *p* < 0.001 vs. control group. ^#^ *p* < 0.05, ^###^ *p* < 0.001 vs. CoCl_2_-treated group.

Pro-inflammatory mediators such as iNOS and COX-2 induce pro-inflammatory cytokines by macrophages in inflammatory response [17]. To explore whether EGCG could affect proinflammatory cytokine expressions in hypoxia-stimulated microglia, the expression levels of IL-6, iNOS, and COX-2 mRNA or protein were determined via real-time PCR analysis or Western blot analysis, respectively, in BV2 cells treated with 350 μM CoCl_2_ for 8 h in the presence of 200 μM EGCG. As shown in Figure 2, CoCl_2_-treated cells dramatically increased mRNA expression of IL-6, iNOS, and COX-2, which were significantly abolished by EGCG. Moreover, EGCG significantly decreased the CoCl_2_-induced expression of IL-6, iNOS, and COX-2 proteins in BV2 cells, as expected. These results demonstrated that EGCG can inhibit inflammatory cytokine production in CoCl_2_-treated BV2 cells.

### 2.3. EGCG Inhibits NF-κB Activity in CoCl_2_-Stimulated BV2 Cells

**Figure 3:**
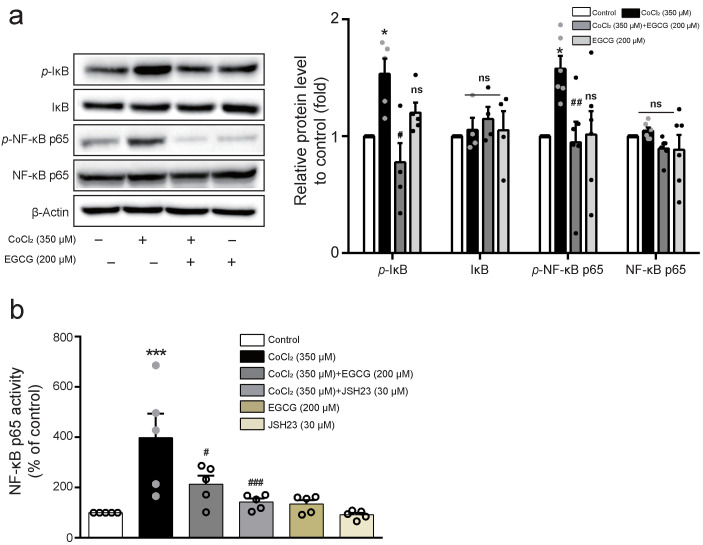
Effect of EGCG on NF-κB activation in CoCl_2_-treated BV2 cells. (**a**) Cells pre-treated with EGCG (200 μM) for 1 h were treated with CoCl_2_ (350 μM) for 8 h. The levels of p-IκB, IκB, p-NF-κB p65, and NF-κB p65 proteins were detected by Western blot analysis and the histograms normalised to β-actin are presented. (**b**) Cells pre-treated with EGCG (200 μM) or JSH-23 (30 μM) for 1 h were treated with CoCl_2_ (350 μM) for 8 h. DNA binding activity of NF-κB p65 was measured from nuclear extracts and quantified by a microplate reader. The data represent mean ± SEM. ns, not significant, * *p* < 0.05, *** *p* < 0.001 vs. control group; ^#^ *p* < 0.05, ^##^ *p* < 0.01, ^###^ *p* < 0.001 vs. CoCl_2_-treated group.

NF-κB p65 is known as a crucial transcription factor mediating pro-inflammatory responses upon exposure to inflammatory signals and mediators controlling many genes [18]. To investigate whether EGCG regulates the activation of NF-κB p65 and the phosphorylation of IκB and NF-κB p65, Western blot analysis was performed in BV2 cells pre-treated with 200 μM EGCG prior to 350 μM CoCl_2_ treatment. Enhanced NF-κB p65 activity following CoCl_2_ treatment was substantially suppressed by the treatment of EGCG in BV2 cells (Figure 3A). Moreover, cells were treated with 200 μM EGCG or 30 μM JSH-23, an inhibitor of NF-κB nuclear translocation and then stimulated with 350 μM CoCl_2_ for 8 h. EGCG or JSH-23 pre-treatment significantly inhibited NF-κB transcriptional activity enhanced by CoCl_2_ treatment (Figure 3B). These results demonstrate that the anti-inflammatory effect of EGCG is associated with the regulation of NF-κB pathway in CoCl_2_ -stimulated BV2 cells.

### 2.4. EGCG Attenuates HIF-1α Expression and ROS Production in CoCl_2_-Stimulated BV2 Cells

**Figure 4:**
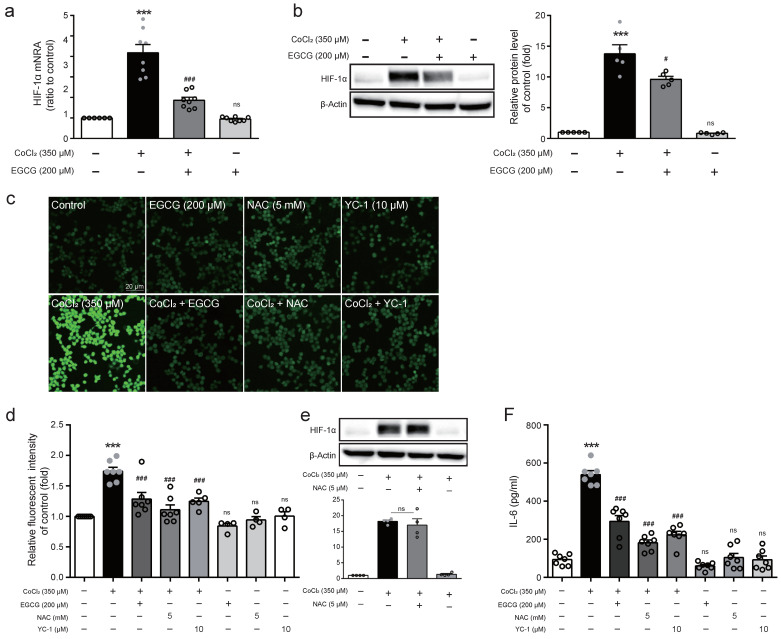
Effect of EGCG on HIF-1α expression and ROS production in CoCl_2_-treated BV2 cells. (**a**,**b**) Cells were treated with or without EGCG (200 μM) for 1 h prior to CoCl_2_ (350 μM) exposure for 8 h and the expression of HIF-1α was determined using real-time PCR and Western blot analysis, respectively. (**c**) ROS generation was determined by H_2_DCFDA and fluorescent images were analysed microscopically in CoCl_2_ (350 μM)-treated cells with EGCG (200 μM), NAC (5 mM), or YC-1 (10 μM). (**d**) Quantitative analysis of DCF fluorescence by microplate reader. (**e**) Cells were treated in the presence of CoCl_2_ (350 μM) with or without NAC (5 mM) for 8 h and the expression of HIF-1α protein levels was detected by Western blot. (**f**) Cells were treated with CoCl_2_ (350 μM) in the presence or absence of NAC or YC-1 for 8 h and the levels of IL-6 protein released in the supernatant were analysed with ELISA. The data represent mean ± SEM. ns not significant, *** *p* < 0.001 vs. control group; ^#^ *p* < 0.05, ^###^ *p* < 0.001 vs. CoCl_2_-treated group.

The expression of HIF-1α, a key factor regulating cellular adaptation to hypoxia [19], was analysed by Western blot in CoCl_2_-treated BV2 cells for 8 h following EGCG pre-treatment for 1 h. The expression of HIF-1α at mRNA and protein levels significantly increased in 350 μM CoCl_2_-treated BV2 cells but was significantly attenuated by 200 μM EGCG pre-treatment (Figure 4A,B). Inflammation can trigger the accumulation of ROS and promote oxidative stress [20]. The generation of ROS, a crucial signaling factor, which plays a critical role in inflammatory disorder, was determined using H_2_DCFDA. As shown in Figure 4C, EGCG suppressed CoCl_2_-induced ROS generation in BV2 cells, similarly to the results of pre-treatment with ROS scavenger NAC. In addition, a co-treatment with NAC or HIF-1α inhibitor YC-1 significantly diminished ROS levels increased by CoCl_2_ (Figure 4D). Furthermore, exposure to NAC prior to CoCl_2_ did not result in a significant suppression of HIF-1α protein levels (Figure 4E), indicating that HIF-1α expression regulates CoCl_2_-induced ROS generation in BV2 cells. Moreover, HIF-1α inhibitor YC-1 significantly reversed IL-6 production increased by CoCl_2_, similarly to the inhibitory effect by NAC (Figure 4F). These results demonstrate that the suppression of ROS production and HIF-1α expression in hypoxia condition represent a crucial mechanism underlying anti-inflammatory action by EGCG.

### 2.5. EGCG Activates Nrf-2 Signaling Pathway in CoCl_2_-Treated BV2 Cells

**Figure 5:**
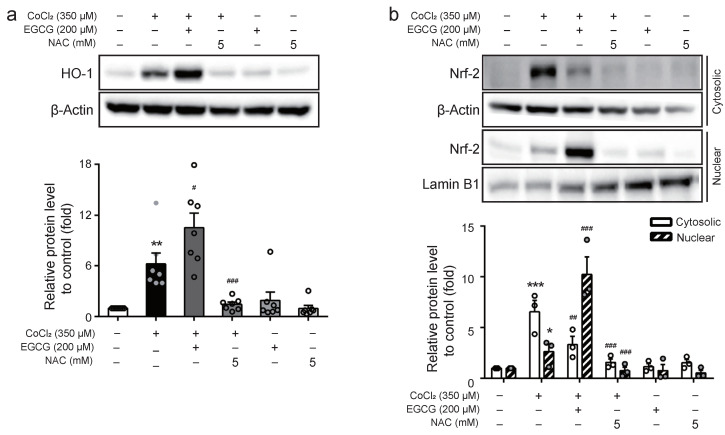
Effect of EGCG on HO-1 expression and Nrf-2 translocation in CoCl_2_-treated BV2 cells. (**a**) Cells were treated with EGCG (200 μM) prior to CoCl_2_ (350 μM) treatment for 8 h, and the protein expression of HO-1 was detected by Western blot analysis. (**b**) The protein levels of Nrf-2 in cytosolic or nuclear fraction were measured by Western blot analysis in CoCl_2_ (350 μM)-treated BV2 cells with EGCG (200 μM) or NAC (5 mM) for 8 h. The results represent mean ± SEM. * *p* < 0.05, ** *p* < 0.01, *** *p* < 0.001 vs. control group; ^#^ *p* < 0.05, ^##^ *p* < 0.01, ^###^ *p* < 0.001 vs. CoCl_2_-treated group.

The oxidative action of LPS-stimulated ROS production is potentially neutralised by antioxidant-related protein HO-1, which is regulated by the transactivation of Nrf-2 [21]. To investigate whether EGCG regulates the Nrf-2/HO-1 pathway in hypoxia conditions, cells were treated with 350 μM CoCl_2_ in the presence of 200 μM EGCG or 5 mM NAC in CoCl_2_-treated BV2 cells for 8 h. Western blot analysis showed that EGCG upregulated HO-1 expression in the presence of CoCl_2_, whereas NAC with or without CoCl_2_ treatment did not regulate HO-1 expression (Figure 5A). Furthermore, CoCl_2_ increased Nrf-2 levels in the cytosolic extract compared with the control, whereas EGCG reversed cytosolic Nrf-2 levels and increased nuclear Nrf-2 compared with the CoCl_2_-treated group. Interestingly, NAC had no effect on Nrf-2 nuclear translocation (Figure 5B). These results suggest that the anti-inflammatory effects of EGCG might be involved in the upregulation of HO-1 via the Nrf-2 pathway in hypoxia-activated BV2 cells.

### 2.6. EGCG Abrogates CoCl_2_-Induced Apoptosis of BV2 Cells

**Figure 6:**
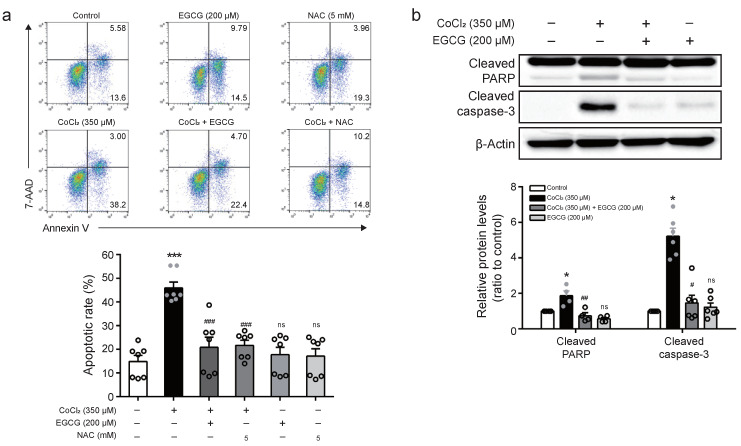
Effect of EGCG on CoCl_2_-induced apoptosis in BV2 cells. (**a**) Cells were treated with EGCG (200 μM) or NAC (5 mM) for 1 h prior to CoCl_2_ (350 μM) for 8 h in BV2 cells. The proportions of apoptotic cells were determined by flow cytometry. (**b**) Cells were treated with EGCG (200 μM) for 1 h prior to CoCl_2_ (350 μM) for 8 h. The levels of cleaved PARP and cleaved caspase-3 were detected by Western blot analysis using anti-PARP and anti-cleaved caspase-3 antibodies. The results are expressed as mean ± SEM. * *p* < 0.05 *** *p* < 0.001 vs. control group; ^#^ *p* < 0.05, ^##^ *p* < 0.01, ^###^ *p* < 0.001 vs. CoCl_2_-treated group.

To investigate whether EGCG suppresses hypoxia-induced apoptosis of BV2 microglia, FACS analysis was performed in the presence of 350 μM CoCl_2_ with 200 μM EGCG or 5 mM NAC. Treatment with EGCG or NAC decreased the rate of apoptosis (%) induced by CoCl_2_ treatment (Figure 6A). Moreover, the cleavages of poly (ADP-ribose) polymerase (PARP) and caspase-3, a prerequisite for cells undergoing apoptosis, were detected by Western blot analysis in CoCl_2_-treated BV2 cells after EGCG pre-treatment. Cleavages of PARP and caspase-3 following exposure to CoCl_2_ in the BV2 cells were increased, while EGCG pre-treatment resulted in reduction in caspase-3 and PARP cleavage, indicating that EGCG suppresses hypoxia-induced apoptosis of BV2 cells (Figure 6B).

## 3. Discussion

Hypoxia is closely related to the occurrence of neuronal diseases, such as stroke, neonatal hypoxic-ischemic encephalopathy, Alzheimer’s disease, Parkinson’s disease, vascular dementia, and epilepsy [22,23,24,25]. Hypoxia leads to M1 microglia, an activated phenotype of macrophage-like cell, which mainly produces pro-inflammatory mediators including TNF-α, IL-1β, and IL-6 and triggers ROS production, possibly inducing a cascade of events leading to neuronal damage [26,27]. Alternatively, M2 microglia facilitate recovery via promoting the release of anti-inflammatory cytokines, such as IL-10 and TGF-β after brain injury [28,29,30]. Therefore, eliminating pro-inflammatory mediators in the neuro-inflammatory response under hypoxic conditions is an important strategy for developing inflammation-related disease therapy. This study investigated the molecular mechanisms involved in the anti-inflammatory effects of EGCG in microglial cells under hypoxic conditions.

Green tea polyphenol EGCG has neuroprotective and immune-protective effects via modulating neuroinflammation [31,32,33]. In a mouse model of Parkinson’s disease, EGCG treatment can effectively reduce the expression of serum inflammatory factors including TNF-α and IL-6 [32]. Moreover, ECG and EGCG significantly reduced the expression levels of TNF-α and IL-1β, and IL-6 was upregulated in lipopolysaccharide-stimulated HDPCs [34]. In addition, EGCG suppressed the release of pro-inflammatory cytokines TNF-α, IL-1β, and IL-6 in microglia under high-fat-induced hypothalamic inflammation [31]. In the present study, BV2 cells in CoCl_2_ treatment induced hypoxia-mimic conditions and increased the expression of inflammatory cytokines IL-6, NOS, and COX-2 in a dose-dependent manner. However, EGCG pre-treatment before CoCl_2_ exposure dramatically decreased CoCl_2_-induced IL-6, iNOS, and COX-2 expressions. These results suggest that EGCG has anti-inflammatory effects in BV microglial cells under hypoxic conditions.

Microglia act the first line of defence and immune response in the central nervous system (CNS) [35]. In particular, damaged microglia produce inflammatory mediators and inflammatory cytokines by regulating the NF-κB pathway in the pathophysiology of inflammatory responses [36,37]. NF-κB p65 is a nuclear transcription factor that regulates the transcription of genes and the activation of inflammatory response [38]. Under normal conditions, NF-κB and IκB bind to form a complex in the cytoplasm. However, pathologic conditions such as LPS or hypoxia result in the degradation of IκB and nuclear translocation of NF-κB p65 from the cytoplasm. Many studies have shown that the NF-κB pathway is associated with diseases related to neuroinflammation. The blockade of NF-κB transcription in murine microglia is known to inhibit the expression of inflammatory mediators such as iNOS and COX-2 and pro-inflammatory cytokines including IL-6 [39,40]. Previous studies reported that NF-κB and HIFs play an important role in activating the expression of genes associated with neo-angiogenesis and inflammatory factors, indicating a close biochemical and functional relationship between inflammation and hypoxia [41,42,43,44]. In the present study, to explore the mechanism underlying the protective effects of EGCG on hypoxia-induced inflammatory response in BV2 microglia cells, we investigated whether EGCG could modulate the activation of NF-κB p65 in CoCl_2_-induced hypoxia. We found that the CoCl_2_-enhanced phosphorylation of NF-κB p65 in the BV2 cells was substantially suppressed by EGCG, leading to a downregulation of iNOS, COX-2, and IL-6 expressions.

Under hypoxic conditions, the accumulation of HIF-1α can activate the transcription of genes associated with oxidative stress and mediates the response of cellular oxygen sensors [45]. As a widely used hypoxic mimic, CoCl_2_ stimulates the upregulation of HIF-1α [46]. In the present study, an exposure of cells to CoCl_2_ upregulated HIF-1α expression, whereas EGCG pre-treatment attenuated the expression of HIF-1α. Hypoxia can also stimulate ROS formation and result in oxidative stress, subsequently leading to many clinical diseases [47]. We found that CoCl_2_-induced ROS production in BV2 cells was significantly reduced by EGCG. In addition, HIF-1α inhibitor YC-1 suppressed ROS overproduction, which was similar to the results of pre-treatment with ROS scavenger NAC, whereas NAC had no effect on HIF-1α expression in CoCl_2_-treated BV2 cells. These findings demonstrated that ROS inhibition via the suppression of HIF-1α signaling mediated the anti-oxidant effect of EGCG against CoCl_2_-induced hypoxia in BV2 cells. Furthermore, oxidative stress enhances microglial inflammatory response, leading to neuronal injury [48,49]. It is known that, during hypoxia, HIF-1α and ROS such as pro-inflammatory factors trigger an inflammatory cascade and are crucial signaling molecules that regulate macrophage activation in hypoxia-stimulated inflammation [50,51,52]. To confirm whether hypoxia-induced HIF-1α and ROS regulate the production of pro-inflammatory factors, treatment with NAC and YC-1 was followed by exposure to CoCl_2_ in BV2 cells. Hypoxia-increased IL-6 production was downregulated by NAC and YC-1 with similar results by EGCG. Our data showed that EGCG abrogated hypoxia-induced inflammatory response by attenuating HIF-1α expression and ROS production in BV2 microglia, consistent with previous studies [53,54,55].

Under normal conditions, Nrf-2 is localised in the cytoplasm and the nuclear translocation of Nrf-2 is inhibited by Keap1. However, under oxidative stress conditions, Nrf-2 is detached from Keap1 and translocated into the nucleus to regulate the expression of antioxidant proteins such as HO-1 [56,57]. Chronic inflammation has been reported in HO-1 deficient models [58]. Meanwhile, it has previously been revealed that ginsenoside Rk1 protects against inflammation and oxidative damage by mediating the Nrf-2/HO-1 signaling pathway [59]. Our results showed that CoCl_2_ treatment decreased the protein levels of nuclear Nrf-2 and HO-1 compared with the untreated group, whereas EGCG promoted the nuclear translocation of Nrf-2 and upregulated HO-1 expression following CoCl_2_ treatment. These results demonstrate that the activation of the Nrf-2/HO-1 pathway is involved in the anti-inflammatory effects of EGCG against hypoxic injury in BV2 microglial cells, similar to previous reports of anti-neuroinflammatory effects of microglia via Nrf-2/HO-1 pathway activation [60,61].

Additionally, EGCG exerts anti-apoptotic activity in various cell or tissue. Previous reports showed that EGCG attenuates the expression of cleaved caspase-3, -8, and -9 in H_2_O_2_-treated vascular smooth muscle cells [62] and inhibits dexamethasone-induced apoptosis in the osteoporosis [63]. Even in the present study, the enhanced rate of apoptosis in BV2 cells exposed to CoCl_2_ was blocked by EGCG or NAC pre-treatment. Furthermore, the constitutive activation of caspase-3 and PARP cleavage induced by CoCl_2_ were ameliorated by EGCG. Not surprisingly, EGCG protected BV2 cells from CoCl_2_-induced apoptosis. These results are consistent with previous work that NAC with strong antioxidant properties prevents H_2_O_2_-induced germ cell apoptosis via the downregulation of caspase-9 and caspase-3 and results in counteracting oxidative stress [64].

The potential health benefits associated with green tea consumption have been partially attributed to the antioxidative property of tea polyphenols, likely due to the radical scavenging effect of EGCG [65]. It has been reported that green tea consumption within a controlled diet can protect against oxidative damage and can prevent chronic diseases in humans [66]. The various pharmacokinetic researches verified that daily intake of 3 to 5 cups/day (720 to 1200 mL) of green tea provides at least 180 mg/day of catechins and may be adequate relative to the range of tea consumption associated with these benefits [67,68]. However, the optimal amount may depend on the disease type or can vary from person to person. Other benefits of natural polyphenols such as EGCG include avoiding gastrointestinal damage of nonsteroidal anti-inflammatory drugs and permeability into the blood–brain barrier can prevent cognitive dysfunction [69,70]. Further studies are needed to define the actual magnitude of health benefits and to establish and elucidate the potential safe mechanisms of action.

## 4. Materials and Methods

### 4.1. Chemicals and Reagents

Fetal bovine serum (FBS) and cell culture media (Dulbecco’s modified Eagle’s medium) (DMEM) were purchased from Hyclone (Logan, UT, USA). Cobalt chloride (C8661) and epigallocatechin gallate (E4143) were purchased from Sigma-Aldrich (St. Louis, MO, USA). The primary antibodies specific for IL-6, iNOS, COX-2, IκB, phosphorylated IκB, NF-κB p65, phosphorylated NF-κB p65, HIF-1α, HO-1, Nrf-2, cleaved caspase-3, and poly-adenosine diphosphate ribose polymerase (PARP) were obtained from Cell Signaling Technology (Beverly, MA, USA), and antibodies targeting Lamin B1, HIF-1α inhibitor YC-1 and NAC were purchased from Santa Cruz Biotechnology, Inc. (Santa Cruz, CA, USA). The ROS detection kit was purchased from Abcam (Cambridge, MA, USA).

### 4.2. Cell Culture

The murine microglial BV2 cell line was cultured in high-glucose DMEM containing 10% FBS and 1% penicillin-streptomycin under humidified conditions and incubated in the presence of 5% CO_2_ at 37 °C. The cells were loaded on a 96-well or 24-well plate at a density of 2 × 10^4^ or 1 × 10^5^ cells/mL.

### 4.3. Cell Viability Assay

Cell viability was measured using an EZ-cytox kit (Dogen, Seoul, Korea). Briefly, cells were seeded on 24-well plates (2 × 10^5^ cell/well) in a humidified atmosphere of 5% CO_2_ at 37 °C. They were treated with different concentrations of EGCG only for 8 h or 1 h before co-treatment with CoCl_2_. Next, each well was treated with EZ-cytox solution and incubated for 30 min followed by a measurement of absorbance at 450 nm using a microplate reader (BioTek Instruments, Winooski, VT, USA) with a reference wavelength of 630 nm.

### 4.4. Quantitative Real-Time PCR

Cells were loaded on 6-well plates at a density of 2 × 10^5^ cells/mL and were pre-treated with EGCG for 1 h prior to CoCl_2_ treatment for 8 h. Extraction of total cellular RNA was performed by treating cells with TRIzol reagent (GeneAll Biotechnology, Seoul, Korea) according to the manufacturer’s instructions. The synthesis of cDNA was accomplished using 1 μg of total cellular RNA using reverse transcriptase (Enzynomics, Daejeon, Korea). Quantitative real-time PCR was performed using the QuantiNova SYBR Green PCR kit (Qiagen, Valencia, CA, USA) and Rotor-Gene 3000 System (Corbett Research, Mortlake, NSW, Australia). The mouse primers used for each gene are listed in Table 1. Single fluorescence reading was recorded for each extension phase. The relative mRNA expression levels were calculated based on the mean cycle threshold (ΔCt) values, and they were normalised to β-actin using Corbett Robotics Rotor-Gene software (Rotor-Gene 6, Version 6.1, Build 90).

### 4.5. Western Blot Analysis

Cells were washed twice with cold PBS after CoCl_2_ treatment for 8 h with or without EGCG for 1 h. Total protein was extracted by a RIPA buffer (Biosesang, Seongnam, Korea) containing a 1% Protein Inhibitor Cocktail and 1% Phosphatase Inhibitor Cocktail (GenDEPOT, Barker, TX, USA). The nuclear and cytoplasmic extracts were obtained using the NE-PER Nuclear and cytoplasmic extraction reagent kit (Thermo Fisher Scientific, Rockford, IL, USA) according to the manufacturer’s instructions. The protein concentration was measured using the BCA protein assay kit (Thermo Fisher Scientific) with Microplate Spectrophotometer (BioTek Instruments). Equal amounts of protein (20~40 μg) were separated by electrophoresis on 8–15% poly-acrylamide gel, followed by transfer to a nitrocellulose membrane for 12 h at 4 °C and blocked with 5% BSA or 5% skim milk (BD Biosciences, Franklin Lakes, NJ, USA) in Tris-buffered saline-0.1% Tween 20 (TBS-T). Next, the membranes were washed three times with TBS-T for 10 min and incubated with diluted primary antibodies (1:1000 dilution in TBS-T) overnight at 4 °C. After washing with TBS-T three times, they were incubated with diluted horseradish peroxidase (HRP)-conjugated secondary antibody (1:20,000 dilution in TBS-T) for 3 h at 4 °C. The membranes were developed using the Western Chemiluminescent HRP Substrate (Millipore, Darmstadt, Germany), and intensity was quantified by using a Fusion FX system with ImageMaster™ assistant software (Vilber Lourmat, Eberhardzell, Germany).

### 4.6. Nuclear Extraction and NF-κB Transcriptional Activity Assay

Cells were pre-treated with EGCG or JSH-23 (an inhibitor of NF-κB nuclear translocation, Millipore) for 1 h and then stimulated with CoCl_2_ for 8 h. Nuclear fractions were extracted using NE-PER™ Nuclear and Cytoplasmic Extraction reagents (Thermo Fisher Scientific). The binding activity of NF-κB p65 to DNA was evaluated using an NF-κB p65 transcriptional factor assay kit (Cayman, Ann Arbor, MI, USA) according to the manufacturer’s instruction. Following transcription factor development, absorbance was read at 450 nm with a microplate reader (BioTek Instruments).

### 4.7. Enzyme-Linked Immunosorbent Assay (ELISA)

Cells were seeded on 6-well plates at 6 × 10^5^ cells per well and incubated with EGCG for 1 h prior to CoCl_2_ treatment for 8 h. Next, 1 mL of the medium was added each well. The concentration of IL-6 in cell culture supernatants was determined via ELISA according to the manufacturer’s instructions (R&D system, Minneapolis, MN, USA). The plates were read at 450 nm and a corrected wavelength of 540 nm using a microplate reader (BioTek Instruments).

### 4.8. Measurement of Intracellular ROS Levels

ROS production was detected using the 2′,7′-dichlorodihydrofluorescein diacetate (H_2_DCFDA) with a Cellular ROS Detection Assay kit (Abcam) following manufacturer’s instructions. Briefly, cells were loaded overnight on 96-well plates at 6 × 10^4^ cells/mL and treated with EGCG or CoCl_2_ for 30 min, followed by incubation with 12.5 μM H_2_DCFDA in 1× dilution buffer for 30 min at 37 °C. The H_2_DCFDA intensity was measured with a microplate reader (BioTek Instruments), and the fluorescence excitation/emission was 485/535 nm. Fluorescent images were captured using a Zeiss LSM900 Airyscan confocal laser scanning microscope with Zen 3.2 software (Carl Zeiss, Jena, Germany).

### 4.9. Apoptosis Assay

Cells plated in 10 cm culture dishes were treated with a high-glucose DMEM medium containing EGCG for 1 h and then cultured with CoCl_2_ for 8 h. To detect apoptotic cell death, cells were stained with Annexin V-PE and 7-AAD-FITC Apoptosis Detection reagent (BD Biosciences) and were analysed via Flow Cytometry (FACSCalibur, BD Biosciences).

### 4.10. Statistical Analysis

Experiments were performed in triplicate at least. Data were expressed as mean ± SEM. They were analysed using one-way analysis of variance (ANOVA) followed by Tukey’s multiple comparison post hoc test according to the normality test results or non-parametric one-way ANOVA (Kruskal–Wallis test) with Dunn’s multiple comparison. Normality was determined via D’Agostino and Pearson omnibus normality test for parametric analyses. All data were analysed with GraphPad Prism 6 Software (GraphPad Software, San Diego, CA, USA).

## 5. Conclusions

Collectively, the results of the present study indicate that EGCG possesses anti-inflammatory and anti-oxidant effects by inhibiting NF-κB pathway and enhancing Nrf-2/HO-1 pathway in CoCl_2_-stimulated BV2 microglial cells, suggesting that EGCG has a protect effect against neuronal damage induced by hypoxia. Finally, our results suggesting neuroprotective efficacy of EGCG against microglial activation and chronic inflammation may provide further support for the clinical trials for the treatment of neuronal disease.

## Figures and Tables

**Table 1:** List of primer sequences used for qRT-PCR analysis.

Gene (Mouse)	Sequence (5′→3′) Used for qRT-PCR
*IL-6*	sense—GATCTGGCACCACACCTTCT
	antisense—GGGGTGTTGAAGGTCTCAAA
*iNOS*	sense—TGACTGTGCACCTACTATGTCACTT
	antisense—GGTCAGCTGTGGTAATCCACTC
*COX-2*	sense—CCACTTCAAGGGAGTCTGGA
	antisense—AGTCATCTGCTACGG GAGGA
*HIF-1* *α*	sense—TCACTGGGA CAGCACAGAAT
	antisense—TGTGTCTGCAGATGTGCTGA
*β* *-Actin*	sense—GCCTTCTTGGGACTGATGCT
	antisense—TGCGGGATCCACACTCTCCAG

## Data Availability

All data collected and analysed during the current study are available from the corresponding author on reasonable request.
